# Uncertainty-based Optimization Algorithms in Designing Fractionated Spacecraft

**DOI:** 10.1038/srep22979

**Published:** 2016-03-11

**Authors:** Xin Ning, Jianping Yuan, Xiaokui Yue

**Affiliations:** 1 College of Astronautics, Northwestern Polytechnical University, Xi’an, Shaan xi 710072, People’s Republic of China; 2 Department of Mechanical & Manufacturing Engineering, Schulich School of Engineering, University of Calgary, Calgary, Alberta, Canada T3A1L9

## Abstract

A fractionated spacecraft is an innovative application of a distributive space system. To fully understand the impact of various uncertainties on its development, launch and in-orbit operation, we use the stochastic missioncycle cost to comprehensively evaluate the survivability, flexibility, reliability and economy of the ways of dividing the various modules of the different configurations of fractionated spacecraft. We systematically describe its concept and then analyze its evaluation and optimal design method that exists during recent years and propose the stochastic missioncycle cost for comprehensive evaluation. We also establish the models of the costs such as module development, launch and deployment and the impacts of their uncertainties respectively. Finally, we carry out the Monte Carlo simulation of the complete missioncycle costs of various configurations of the fractionated spacecraft under various uncertainties and give and compare the probability density distribution and statistical characteristics of its stochastic missioncycle cost, using the two strategies of timing module replacement and non-timing module replacement. The simulation results verify the effectiveness of the comprehensive evaluation method and show that our evaluation method can comprehensively evaluate the adaptability of the fractionated spacecraft under different technical and mission conditions.

With the rapid development of computational capability, sensor technology and communication technology, the traditional monolithic spacecraft tends to be coordinated and it was controlled by multiple spacecraft whose control center is network. In order to solve the personal, technical, environmental, launch, demand and funding uncertainties’ problem etc, Charlotte and Annalisa from the Massachusetts Institute of Technology came up with a completely new concept of fractionated spacecraft in 2005^1^. We attempt to further explain the concepts of constellation, formation and fractionated spacecraft.

## Constellation

It is a satellite system that including several satellites that operate on an orbit plane or multi-orbit plane. A constellation is made up of *n* number of orbit planes, and there are *m*_*i*_ satellites on the *i*(1 ≤ *i* ≤ *n*)th plane. The construction of the constellation can be described as follows:


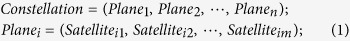


Among them, *Plane*_*i*_ denotes the *i*th orbit plane; *Satellite*_*ij*_ denotes the *j*(1 ≤ *j* ≤ *m*)th satellite on the *i*th orbit plane.

## Formation

It shows how satellites are networked. Several small satellites that are moving around the earth in their own orbits also keep their own fixed phases and distances; in other words, they move in a certain formation, which is usually an inscribed polygon. The construction of formation can be described as follows:


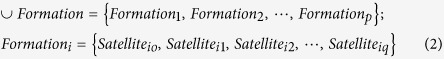


Among them, *Formation* denotes the *i*(1 ≤ *i* ≤ *p*)th simple formation, *Satellite*_*ij*_ denotes the reference satellite in the *i*th simple formation, and *Satellite*_*ij*_ denotes the *j*(1 ≤ *j* ≤ *q*)th circling satellite in the *i*th simple formation.

## Fractionated Spacecraft

The separation of the traditional monolithic spacecraft which makes it into several separate modules that can be combined to form a virtual integration architecture with regular functions, which is connected through wireless data and transmitted with wireless energy in orbit.

The structure of a fractionated spacecraft can be described as:


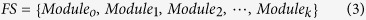


Among which, *Module*_*o*_ denotes the reference module or virtual reference module of the fractionated spacecraft; *Module*_*i*_ denotes the *i*(1 ≤ *i* ≤ *k*)th circling module of the fractionated spacecraft.

Thus, the fractionated spacecraft is different from satellite constellation and formation; it is not a combination of several conventional satellites that have complete functions. In fact, it is a formation of spacecraft with fractionated modules (for example, independent power source module, effective payload module, communication module), thus having strong maneuverability and flexibility.

The concept of fractionated spacecraft attracted the attention of the AFRL (Air Force Research Laboratory) and the DARPA (Defense Advanced Research Projects Agency)^2^,^3^. In September of 2007, on the basis of preliminary study and taking into account the operationally responsive space (ORS) which was recruited by the American army, the DARPA proposed to develop the F6 (Future, Fast, Flexible, Free-Flying, Fractionated) system, and invested a huge amount of cost to promote it^4^,^5^,^6^.

The advantages of the fractionated spacecraft in survivability, flexibility, responsiveness, operational lifetime and low cost lying in its ability to flexibly reduce the impact of uncertainties in research, launch and in-orbit operation flexibly, also in enhancing its efficiency and reducing its risks and costs. In order to evaluate the new kind of spacecraft comprehensively, one effective way is to compare the stochastic missioncycle cost of the traditional spacecraft with the fractionated spacecraft which with various configurations under uncertainties.

We systematically describe its concept and then analyze its evaluation and optimal design method that exists during recent years and propose the stochastic missioncycle cost for comprehensive evaluation. We also establish the models of the costs such as module development, launch and deployment and the impacts of their uncertainties respectively. Above all, we carry out the Monte Carlo simulation of the complete missioncycle of various configurations of the fractionated spacecraft with under various uncertainties, give and compare the probability density function and statistical characteristics of its stochastic missioncycle cost, using the two strategies of fixed time replacement and no-fixed time replacement. The results of simulation verify the effectiveness of the comprehensive evaluation method, also show that our evaluation method can comprehensively evaluate the adaptability of the fractionated spacecraft under different technical and mission conditions.

## Result

### Stochastic missioncycle cost analysis model

The evaluation of the flexibility, rapid response, performance robustness of a fractionated spacecraft needs the evaluation of its architectures. Relevant literature can be traced back to 1984; Molette evaluates the performances of a cluster satellite and an in-orbit assembled large platform respectively and concludes that the cluster satellite is not as effective as the in-orbit assembled platform^7^. However, the conclusion is inappropriate, because cost of it is its prerequisite and no quantitative analysis of adaptability, flexibility and development potential and other factors is made. Rooney studied how to divide the separation module of a communications satellite and held that the efficiency of module separation depends on the type of mission; from the perspective of cost, the geostationary orbit communications mission is not suitable for module separation^8^. Charlotte and Annalisa systematically evaluated the topological structure of a fractionated spacecraft and proposed the quantitative evaluation method for multi-attribute design space exploration, which is a method of user-centered and considers the maintainability, scalability, flexibility and rapid response^9^. Brown and Eremenko introduces a value-centered design method into the design of a fractionated spacecraft^10^. Its basic idea is to transform the fractionated spacecraft design problem into the optimization problem. Jarret and Joseph has developed a comprehensive analysis tool of the topological structure of the separation module of the fractionated spacecraft, which was called the GT-FAST (Georgia Tech F6 architecture synthesis tool), and applied it to the exploration of the topological design space of the System F6^11^. Brown proposes the evaluation indicators of the full lifecycle cost under uncertainties^12^. The indicators treats the full lifecycle costs as stochastic variables and are used to do the Monte Carlo simulation of the lifecycle of the fractionated spacecraft under various uncertainties, thus evaluating its robustness, flexibility, responsiveness and so on^13^,^14^. Their values are converted into currency values and integrated into its lifecycle costs. From those above, we obtain the distribution characteristics of its lifecycle costs^15^.

In order to fully compare the advantages with its disadvantage of different configurations, the most important thing is to establish the space mission cost model that accounts the impact of uncertainties within the whole missioncycle, that is to say the lifecycle cost of the fractionated spacecraft should make the missioncycle costs of development, launch, deployment, operation, maintenance, replacement and so on into consideration, obtaining the comprehensive evaluation results through their distribution characteristics. The essential work here is to establish the cost model of the fractionated spacecraft.

### The fractionated spacecraft’s cost model

**Figure 1:**
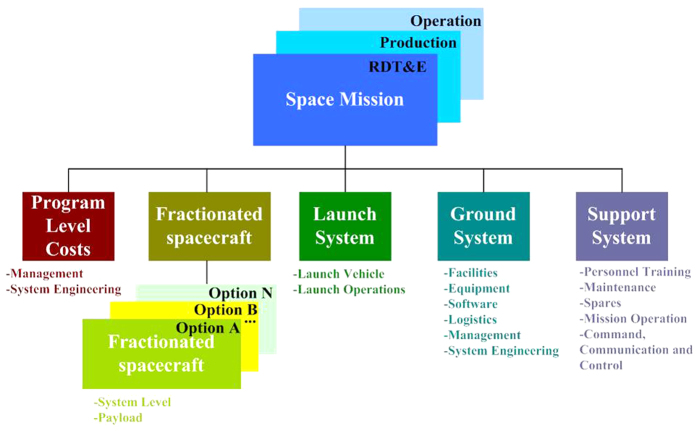
The classification of costs.

The cost model includes the research, development, test & evaluation (RDT&E) of the fractionated spacecraft and its production, launch and operation^16^ (see the Fig. 1).

### The module development cost

We use a small satellite’s module cost estimation model to estimate the Theoretical First Unit (TFU) cost. The Nth repetitive development cost equals to the initial development cost multiplied by the factor of awareness curve:


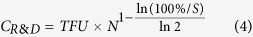


where 
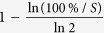
 is the learning rate of the module development and production, *S* is the percentage slope rate of the awareness curve (the value here is 95%).

A traditional spacecraft uses the mature technology; if the application of new technologies increases the cost, then we use the cost correction coefficient to correct it.

### Launch and operation cost

The launch and operation cost of a traditional spacecraft is the single launch and operation cost.

Due to the variation in its carrying capacity, the launch and operation cost of a fractionated spacecraft increases same as the increasing number of modules. In establishing the launch cost model, it is necessary to reduce not only the launch cost but also the times of launch. Therefore, we establish the objective function to contain both of launch cost and launch times.

Let us assume that the *n* number of modules of a fractionated Spacecraft needs to be carried to its orbit, the mass of every module is *m*_*i*_(*i* = 1, 2, …, *n*), *M* types of carrier rockets can be used and the carrying capability of each type is *v*_*j*_(*j* = 1, 2, …, *M*), every rocket has a fixed launch cost *c*_*j*_ and a changeable cost of residue mass per unit (the carrying capacity that is not used for carrying payloads to the orbit) *a*_*j*_. In this case, the mathematical model is as follows:

The objective function:


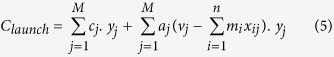


where the two judgment variables are:

If module *i* is carried by the carrying rocket *j*, then *x*_*ij*_ = 1, else *x*_*ij*_ = 0.

If the carrying rocket *j* is not empty, then *y*_*j*_ = 1, else *y*_*j*_ = 0.

We define the operation cost of every module as approximately 2MS.

### Establishing the cost model for uncertainties

We extract the uncertainties existing because of the changes in cost of the design, launch, delivery, operation, maintenance and replacement and establish their models. We treat all these uncertainties as stochastic variables and model them with the probability method.

### Establishing the cost model for uncertainty in developing modules

Its essence lies in the probability distribution of faults in the time domain of every module from its development to the end of its life cycle and its replacement. The faults of a module include repairable fault and irreparable fault. We use the Weibull distribution as the probability distribution of the module’s uncertainties^17^,^18^.

For irreparable fault, the model of life *t*_*R*_ of a given module with the reliability *R* is


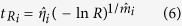


where 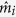 and 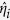 are the estimated values for the Weibull distribution’s parameters *m, η*.

Maintenance needs to be done in case of reparable fault. Assume that the fault occurs when the module works at the time point *t* and that its residue life is denoted by stochastic variable *X*, then after basic repairing, due to damage accumulation, the residue life is distributed as


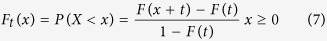


Given the stochastic number *r* in the interval (0, 1), the sampling formula of the residue life is


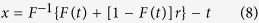


### Establishing the cost model for uncertainty in launch and deployment

We treat the failure of launch and deployment as a point-probability event and assume that the typical probability for orbit entry is 93.9% and that the launch failure, orbit entry failure and initialization failure account for 2.0%, 3.1% and 1.0% respectively.

### Simulating the missioncycle cost under uncertainty

We take the stochastic missioncycle cost under uncertainty as the criteria for assessing the stability, flexibility, responsiveness and many other properties of the fractionated spacecraft to carry out the Monte Carlo simulation of the missioncycle, so as to obtain the distribution characteristics of the missioncycle cost.

According to the subsystem configurations of the traditional spacecraft, we extract and separate the modules as follows: the payload module (abbreviated as P/L), communication system module (abbreviated as C), control and data processing module (abbreviated as D), the power supply module (abbreviated as PW), propulsion system module (abbreviated as P), attitude determination and control module (abbreviated as A).

**Figure 2:**
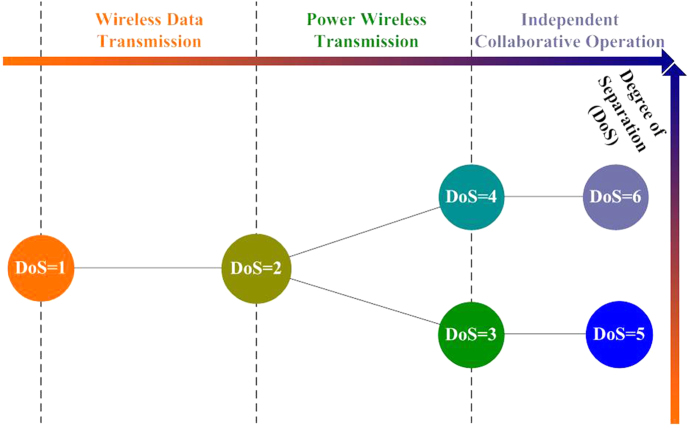
The fractionated spacecraft’s configurations with different DoS.

With increasing Degrees of Separation (abbreviated as DoS) of the fractionated spacecraft, it transmits the wireless data within its clusters, wireless electricity and operates with independent collaboration and other new technologies. According to this, we try to design the various configurations with high feasibility and simulate and verify the fractionated spacecraft evaluation methods (see Fig. 2 below).

Where DoS = 1, DoS = 2, DoS = 3, DoS = 4, DoS = 5 and DoS = 6 stand for 6 kinds of fractionated spacecraft respectively.

DoS = 1: P/L + C + D + PW + P + A

DoS = 2: P/L C + D + PW P + A

DoS =3: P/L C + D PW P + A

DoS = 4: P/L C + D PW P A

DoS = 5: P/L C D PW P + A

DoS = 6: P/L C D PW P A

Table 1 gives the main parameters of the separable subsystems of the fractionated spacecraft. According to the parameters, we can estimate the costs of different configurations and then correct them with the state correction coefficients. Because the traditional spacecraft uses the currrently existing and basically mature technology, its correction coefficient is 0.3, whereas the various configurations of fractionated spacecraft choose different correction coefficients according to the new technology it uses.

## Discussion

The simulation assumes the missioncycle is 30 years, the typical probability for orbit entry is 93.9%, among which the launch failure is 2.0%, the orbit entry has 3.1% failure, the initialization failure is 1.0%, some of electronic circuit component failures can be repaired, the modules obey the Weibull distribution. Above those assumptions, we analyze the stochastic missioncycle costs of the fractionated spacecraft under certainty with the two strategies of fixed time module replacement and offtime module replacement.

### Fixed time module replacement

**Figure 3:**
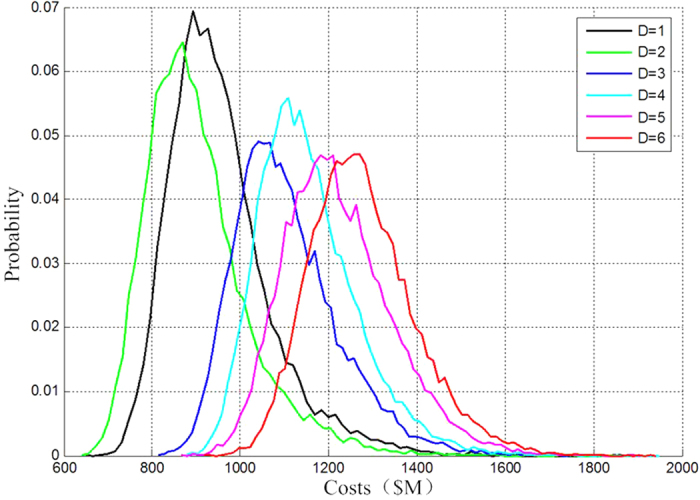
The stochastic missioncycle cost probability density function and their statistical characteristics.

This refers to the replacement of modules according to the average life expectancy obtained by several times’ Monte Carlo sampling, instead of single-time sampling. With 30 years as the missioncycle and 200000 times of simulation, the stochastic missioncycle cost probability density function and their statistical characteristics of the traditional spacecraft and five configurations of fractionated spacecraft as shown in Fig. 3.

Table 2 gives the mean values of stochastic missioncycle costs and the standard deviations of cost probability density function of various configurations.

**Figure 4:**
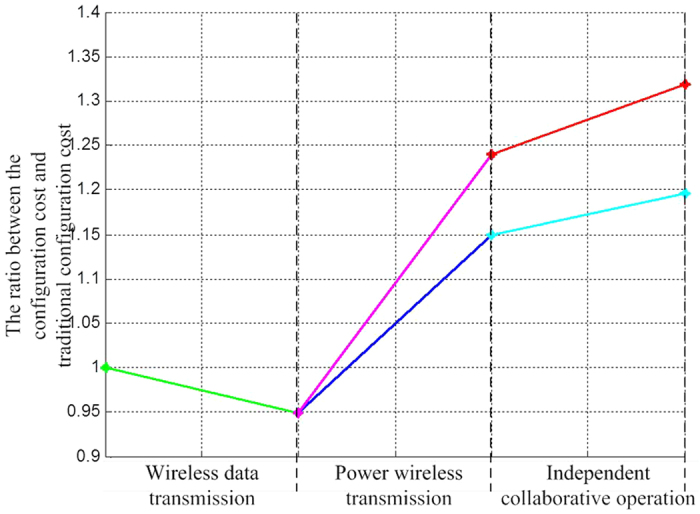
The proportional relationship between the mean values of costs of various configurations and those of the traditional configurations.

The proportional relationship among the mean values of costs of various configurations and those of traditional configurations and the use of new technologies like wireless data transmission, power wireless transmission and independent collaborative operation are shown in Fig. 4.

The simulation draws the conclusions as follows:

- The stochastic missioncycle costs of various configurations of fractionated spacecraft increase with the numbers of separation modules were increased. This is partly because the total cost of a fractionated spacecraft does not completely depend on its reliability. The costs for the development and technical innovation of separation modules increase with their increasing numbers, whereas the separation modules are still subject to limited lower limits of average life expectancy.
- The reason why Configuration DoS = 2 costs less than the traditional spacecraft is that the intermodule communication and the upper level technology are mature, thus costing less to launch, having higher reliability and being able to response rapidly.
- Before the autonomous and cooperative operation technology of a distributed satellite system was implemented, because degradation products were not been used, massive and complex DMS, power, communication, attitude determination, control and propulsion subsystems increased the total mass of a spacecraft, thus lowering down its reliability and affecting its missioncycle cost to a great extent. This fully indicates that the use of the technology is very important for designing a fractionated spacecraft.
- The increasing number of separation modules increases the mass and power consumption of the fractionated spacecraft. When DoS ≥ 3, the costs of stochastic missioncycle various configurations are higher than those of traditional spacecraft. Although their increase is not significant, this also proves the flexibility, high reliability, rapid response and other characteristics of fractionated spacecraft.

### Offtime module replacement

**Figure 5:**
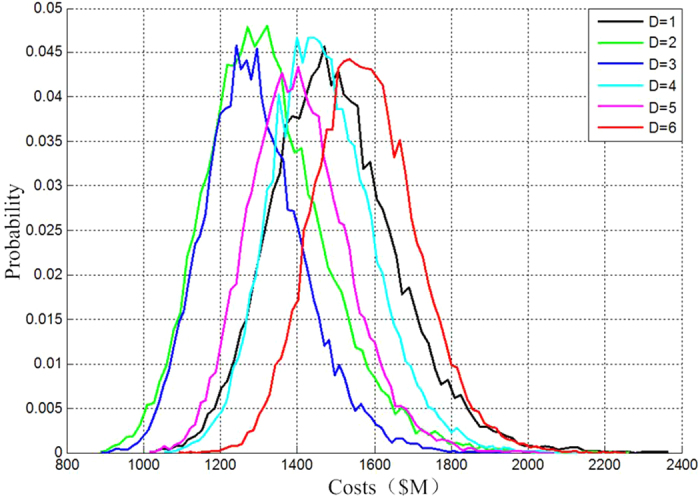
The stochastic missioncycle cost probability density function and their statistical characteristics.

This refers to the replacement of modules according to the average life expectancy obtained by a single time of the Monte Carlo sampling. With 30 years as the missioncycle and 200000 times of simulation, we obtain the stochastic missioncycle cost probability density function and their statistical characteristics of the traditional spacecraft and five configurations of a fractionated spacecraft as shown in Fig. 5.

Table 3 gives the mean values of stochastic missioncycle costs and the standard deviations of cost probability density function of various configurations.

**Figure 6:**
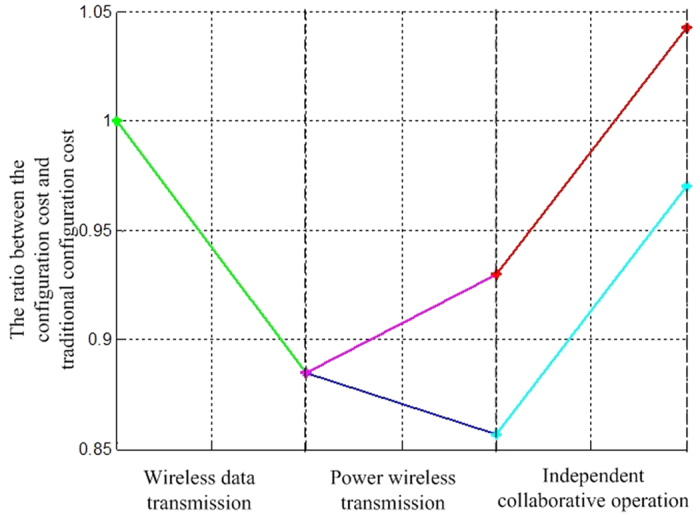
The proportional relationship between the mean values of costs of various configurations and those of the traditional configurations.

The proportional relationship between the mean values of costs of various configurations and those of the traditional configurations and the use of new technologies like wireless data transmission, power wireless transmission and independent collaborative operation are shown in in Fig. 6.

We draw the following conclusions through simulation:

- Except the DoS = 6 configuration, the stochastic missioncycle costs of other configurations are smaller than those of traditional spacecraft’s configurations; with offtime module replacement, the separation module is not constrained by the lower limit of average life expectancy, and the increasing number of separation modules increases the maneuverability of the fractionated spacecraft, reduces its emergency manufacturing cycle and launch cost. The high costs for technical development and module manufacturing of the DoS = 6 configuration are the major cost items among other costs.
- The cost for the DoS = 2 configuration is greater than that of the DoS = 3 configuration because there is no lower limit constraint of the average life expectancy, smaller emergency manufacturing cycle and launch cost. These factors reduce the development and technological innovation costs. The increasing number of modules for the DoS = 4 configuration and the separation of the propulsion module from the attitude determination module contribute to the further increase of development and technological innovation costs, which exceed the costs of the DoS = 2 configuration.
- The cost of the DoS = 5 configuration cost is larger than that of the DoS = 4 configuration. Although both have the same number of modules, the latter’s application of autonomous operation collaboration technology reduces the costs for ground equipment, personnel, monitoring control and communication greatly.

## Additional Information

**How to cite this article**: Ning, X. *et al.* Uncertainty-based Optimization Algorithms in Designing Fractionated Spacecraft. *Sci. Rep.* **6**, 22979; doi: 10.1038/srep22979 (2016).

## Figures and Tables

**Table 1:** The main parameters of the separable subsystems.

Subsystem	Mass (kg)	Power(W)	TFU cost ($M)
D	31.5	28.1	4.428
A	83.8	155.1	7.229
PW	41.3	10.0	1.118
P	88.9	4.7	1.34
C	71.5	228.3	12.516
PL	129.0	188	45.294
Structure/Thermal control	236.9	221.4	3.226
Total	682.9	835.6	75.151
Project Management ($M)			27.054
Ground equipment ($M)			29.764

**Table 2:** The mean values of stochastic missioncycle costs and the standard deviations of cost probability density function of various configurations.

Configuration	Mean values of cost ($M)	Standard deviation ($M)
DoS = 1	1014.3467	7.1513
DoS = 2	962.413	6.7918
DoS = 3	1166.3702	8.4233
DoS = 4	1212.8546	9.2465
DoS = 5	1258.114	9.4763
DoS = 6	1338.39	10.2346

**Table 3:** The mean values of stochastic missioncycle costs and the standard deviations of cost probability density function of various configurations.

Configuration	Mean values of costs($M)	Standard deviation($M)
DoS = 1	1581.9516	8.8736
DoS = 2	1400.0321	8.0734
DoS = 3	1355.3606	9.7094
DoS = 4	1535.1083	10.7326
DoS = 5	1471.3217	10.5737
DoS = 6	1649.1389	11.7077
